# Encapsulation of *E. coli* phage ZCEC5 in chitosan–alginate beads as a delivery system in phage therapy

**DOI:** 10.1186/s13568-019-0810-9

**Published:** 2019-06-17

**Authors:** Abdallah S. Abdelsattar, Fatma Abdelrahman, Alyaa Dawoud, Ian F. Connerton, Ayman El-Shibiny

**Affiliations:** 1 Center for Microbiology and Phage Therapy, Zewail City of Science and Technology, October Gardens, 6th of October City, Giza, 12578 Egypt; 2 Center for X-Ray and Determination of Structure of Matter, Zewail City of Science and Technology, October Gardens, 6th of October, Giza, 12578 Egypt; 3 School of Biosciences, University of Nottingham, Sutton Bonington Campus, Loughborough, UK; 4 Faculty of Environmental Agricultural Sciences, Arish University, North Sinai, Arish, Egypt

**Keywords:** *E. coli*, Bacteriophage, Biocontrol, Phage encapsulation

## Abstract

Bacteriophages can be used successfully to treat pathogenic bacteria in the food chain including zoonotic pathogens that colonize the intestines of farm animals. However, harsh gastric conditions of low pH and digestive enzyme activities affect phage viability, and accordingly reduce their effectiveness. We report the development of a natural protective barrier suitable for oral administration to farm animals that confers acid stability before functional release of bead-encapsulated phages. *Escherichia coli* bacteriophage ZSEC5 is rendered inactive at pH 2.0 but encapsulation in chitosan–alginate bead with a honey and gelatin matrix limited titer reductions to 1 log_10_ PFU mL^−1^. The encapsulated phage titers were stable upon storage in water but achieved near complete release over 4–5 h in a simulated intestinal solution (0.1% bile salt, 0.4% pancreatin, 50 mM KH_2_PO_4_ pH 7.5) at 37 °C. Exposure of *E. coli* O157:H7 to the bead-encapsulated phage preparations produced a delayed response, reaching a maximal reductions of 4.2 to 4.8 log_10_ CFU mL^−1^ after 10 h at 37 °C under simulated intestinal conditions compared to a maximal reduction of 5.1 log_10_ CFU mL^−1^ at 3 h for free phage applied at MOI = 1. Bead-encapsulation is a promising reliable and cost-effective method for the functional delivery of bacteriophage targeting intestinal bacteria of farm animals.

## Introduction

Antibiotic resistance is a serious public health problem worldwide. Commercially available antibiotics are becoming less effective as resistance rates rise over time (Akinkunmi and Lamikanra 2015). Accordingly, many intestinal bacterial infections are showing greater virulence and/or persistence (Munot and Kotler 2016). Such resistance phenotypes are generally attributed to the misuse of antibiotics, which have increased invulnerability to hamper the treatment of infection, and indirectly increase the rate of mortality. Antibiotic use and resistance presents a real dilemma for developed and developing countries (Fortini et al. 2011; Pavlickova et al. 2015; Wellington et al. 2013).

Enterohemorrhagic *Escherichia coli* O157:H7 is a zoonotic pathogen frequently isolated from healthy cattle and other farm animals. The organism causes human gastroenteritis, haemorrhagic colitis, and can lead to the development of hemolytic uremic syndrome (Karmali et al. 1983). Isolates often show multi-drug resistant phenotypes with reports indicating resistance to 14 different antibiotics (Verstraete et al. 2013). *E. coli* O157:H7 can be acquired from direct contact with infected animals (Belongia et al. 1991), or through cross-contamination of raw materials in the preparation of foods, or through the consumption of contaminated food (Neil et al. 2012). *E. coli* O157:H7 remains a threat to public health.

Bacteriophages represent an alternative treatment for the control of bacterial contamination in foods as well as the control of bacterial infections in man and animals due to their abilities to specifically target bacterial host cells and self-replicating nature (Jassim and Limoges 2014; Summers 2001; Taha et al. 2018). Research has demonstrated the use of bacteriophages to reduce *E. coli* O157:H7 in the gastrointestinal tracts of mice (Tanji et al. 2005) and sheep (Bach et al. 2003; Raya et al. 2011), and on the surface of the meat (El-Shibiny et al. 2017; O’Flynn et al. 2004). Studies also suggest phage application could decrease the mortality rate of poultry on infected farms (Xie et al. 2005).

The oral application of phage in human trials has not reported any adverse effects (Bruttin and Brussow 2005; Sarker et al. 2012; McCallin et al. 2013). However, the oral application of phage is not without difficulty due to exposure to gastric juice (GJ) during stomach transit, which may affect the viability of bacteriophages (Tóthová et al. 2012). In light of the above, phage encapsulation techniques have provided a protective delivery technique for phage against the harsh conditions of GJ with minimal phage loss (Choińska-Pulit et al. 2015). Previous publications have highlighted the possibility of using food-grade alginate and chitosan as biomaterials for the microencapsulation of bacteriophages (Ma et al. 2008, 2012; Tang et al. 2013; Kim et al. 2015; Colom et al. 2017). Alginate is considered a good system for phage encapsulation because of its ability to resist acidity, and to control and sustain the release of live products to the gut such as probiotic bacteria and bacteriophages (Gbassi et al. 2009; Lee and Heo 2000). Alginate polysaccharide can be obtained naturally from bacteria and algae, which crosslinks to form a gel with calcium (Lee and Heo 2000). Chitosan is a natural polymer that can be obtained from crustaceans with inherent bacteriostatic and antifungal properties (Mcknight et al. 1988). Accordingly, it is inappropriate for use as a core solution for capsules (Sudarshan et al. 1992), but can be used as a coating material in pharmaceutical applications due to its solubility in acid conditions coupled with excellent biodegradable and biocompatible properties (Allan et al. 1984). Retention of the bead structure and preservation of the phage payload requires that the inner matrix have suitable aqueous viscosity. To this end formulations with gelatin to improve the functional properties of the beads (Gbassi and Vandamme 2012), and honey to stabilize the phage (Oliveira et al. 2017) were explored. In general, the encapsulation process could protect phages against harsh conditions such as acidity and oxidation, control of the release of the active agents, facilitate their diffusion and improve effectiveness (Ghosh et al. 2006; Jyothi et al. 2010; Tang et al. 2013). The objective of this study was to develop a stable chitosan–alginate bead delivery system for the controlled release of bacteriophages. We have examined the protection afforded by the beads for *E. coli* O157:H7 bacteriophages under simulated GI conditions and storage conditions with respect to retention of bacteriophage titers. We demonstrate that the beads are an effective delivery agent for phage with advantages in reducing *E. coli* O157:H7 viable counts under simulated intestinal conditions.

## Materials and methods

### Bacterial strain and culture conditions

Studies were conducted using the bacterial host *E. coli* O157:H7 NCTC 12900 (the kind gift of Dr. Elizabeth Kutter). Bacteriophage were routinely propagated on *E. coli* O157:H7 NCTC 12900. Stocks were maintained in 20% (v/v) glycerol at − 80 °C. In the following experiments, bacterial strains were grown on tryptic soy agar (TSA; Oxoid, England) overnight and infections carried out in Tryptic Soya Broth (TSB; Oxoid, England) in Erlenmeyer flasks at 37 °C and 120 RPM to reach OD600 approximately 0.3.

### Bacteriophage isolation and enumeration

Bacteriophages were isolated by us from environmental and sewage samples against *E. coli* O157:H7 NCTC 12900. Each sample (~ 1 mL) was mixed with TSB containing the bacterial host and incubated overnight at 37 °C to amplify any available phage. After incubation, each sample was serially diluted and spotted on to bacterial lawns of *E. coli* O157:H7 NCTC 12900 to identify any bacteriophages by checking the production of plaques in the bacterial lawn by the 2nd day. A single plaque from a positive agar plate was purified by repeated single plaque isolation using sterile micropipette tips (Adams 1959). All isolated bacteriophages were amplified in TSB and the lysate was centrifuged at 6400×*g* for 15 min at 4 °C to remove the bacterial cells and debris (Marcó et al. 2012). The supernatant was then centrifuged at 15,300×*g* at 4 °C for 1 h to obtain the precipitated pellet of bacteriophages. Bacteriophage pellets were re-suspended in SM buffer (100 mM MgSO_4_·7H_2_O; 10 mM NaCl; 50 mM Tris-HCl pH 7.5) and filtered using 0.22 μm syringe filters (Chromtech, Taiwan). The purified bacteriophage stock was then enumerated as plaque-forming unit (PFU) using double-agar overlay plaque assays (Kropinski et al. 2009), and stored in SM buffer at 4 °C prior to use (Lillehaug 1997). The phage isolate ZCEC5 used in this study can be obtained from Biomedical Sciences Program, Zewail City of Science and Technology, 12578 Giza, Egypt.

### Characterization of bacteriophage ZCEC5

Bacteriophage ZCEC5 was examined using transmission electron microscopy at the National Research Center (Cairo, Egypt) as previously described (Atterbury et al. 2003). Briefly, fixed phages on Pioloform grids using glutaraldehyde were negatively stained with 0.5% uranyl acetate. After drying, the specimens were examined using a JEOL 100CX transmission electron microscope.

Genomic DNA was extracted from a lysate of phage ZCEC5 (10^10^ PFU mL^−1^) treated with proteinase K (100 μg mL^−1^ in 10 mM EDTA at pH 8) before purification by the Wizard DNA kit (Promega, UK) according to the manufacturer’s instructions. The genome DNA of phage ZCEC5 was sequenced from libraries prepared using the Illumina tagmentation protocol on the MiSeq platform. The data was composed of 0.52 million paired-end sequence reads with read lengths of approximately 250 bp. The data was de novo assembled using CLC Genomics Workbench version 10.0.1 (Qiagen, Aarhus, Denmark). The open reading frames (ORFs) were predicted from PHASTER (Arndt et al. 2016). The genome DNA sequence appears in GenBank under the Accession Number MK542015.

### Encapsulation of bacteriophages

**Fig. 1:**
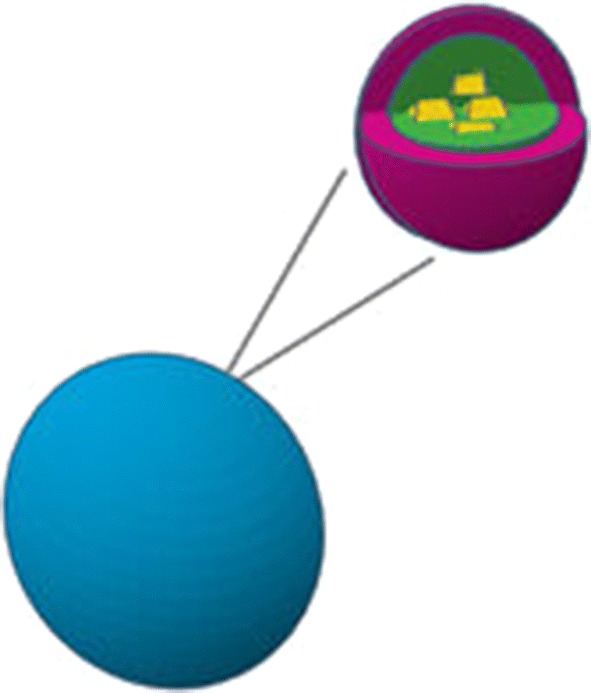
Representation of the bead encapsulation components in cross-section. The blue color refers to the chitosan, purple the Ca-alginate, green the internal matrix and yellow represents the bacteriophage

Encapsulated bacteriophages were prepared using a chitosan–alginate coating shell (Fig. 1). Four matrices were prepared to produce the beads for study. The matrices for beads 1 and 2 were prepared by suspending bacteriophages in either 0.3% commercial honey and 0.25% gelatin or 3% honey to 2.5% gelatin, respectively. The matrix for bead 3 was prepared by suspending bacteriophages in 50 mM Tris-HCl pH 7.4, while the matrix for beads 4 was prepared by suspending bacteriophages in 0.01% gelatin, 0.05% honey, 0.15 M NaCl and 10 mM MgSO_4_·7H_2_O. Each type of matrix was mixed with 1.5% sodium alginate and then extruded into a 100 mM CaCl_2_ solution using a syringe before it was washed with distilled water after 30 min. The prepared Ca-alginate beads were coated with chitosan applied in a chitosan (0.4%)-acetate (100 mM) buffer solution (pH 4.2) for 30 min. The beads were washed with distilled water and stored at 4 °C prior to use.

### Bacteriophage stability and release under simulated intestinal conditions

The stability of encapsulated phages in simulated intestinal conditions was tested by preparing an artificial intestinal juice by dissolving 0.1% bile salt and 0.4% pancreatin (Sigma-Aldrich, MO, USA) in 50 mM KH_2_PO_4_ pH 7.5 (Kim et al. 2015). The beads of encapsulated bacteriophages at 2 × 10^7^ PFU mL^−1^ were incubated in simulated intestinal juice for 6 h at 37 °C with agitation. The free bacteriophage titer was determined using double-agar overlay plaque assays as described above.

### Acid stability assay

The stability of encapsulated bacteriophages (beads) at the digestive system pH ranges was evaluated in 0.5% NaCl solution adjusted to different pH values (2, 2.5, 3, 4 and 7) by the addition of 1 M HCl solutions. Beads were incubated in solutions of various pH for 60 min at 37 °C. After washing with distilled water, beads were incubated at 37 °C for 60 min in a dissolving buffer solution (50 mM sodium citrate, 0.2 M sodium bicarbonate and 50 mM Tris-HCl at pH 7.3) (Liu et al. 2002), and the titers of the released bacteriophages determined using the double-agar overlay plaque assays.

### Thermal stability assay

The stability of encapsulated and non-encapsulated bacteriophages over a range of temperatures was evaluated by incubating phage suspensions in SM buffer at 25, 40, 60 and 80 °C for 60 min. To detect the protective effect of matrices against thermal conduction, the encapsulated and non-encapsulated bacteriophage were exposed to 80 °C and samples were taken at 0, 30, 180 s intervals to detect the change in phage titer upon sudden temperature alteration. The titers of released bacteriophages were determined using the double-agar overlay plaque assays.

### Examination of bead morphology

Encapsulated bacteriophages samples were investigated using a Trinocular Zoom Stereo microscope (Meiji Techno, EMZ-13TR).

### Diffusion properties of stored encapsulated bacteriophages

Encapsulated bacteriophages were stored in flasks containing 200 mL of distilled water at 4 °C. Samples of water were collected at various time points to determine the phage titers released using double-agar overlay plaque assays.

### Lytic activity assay

Encapsulated and non-encapsulated bacteriophage were tested for their lytic activity against *E. coli* O157:H7 NCTC 12900 by incubating each type of beads with *E. coli* in intestinal buffer at 37 °C with agitation at 120 rpm. The infection was performed at MOI = 1 and samples were collected after 3, 6 and 10 h of incubation for analysis.

### Statistical analysis

All statistical analyses were carried out in triplicates. In this study, the Student’s t-test and one-way ANOVA were used as statistical analysis test. The significance level was *p* < 0.05. Data were analyzed using GraphPad PRISM version 5.01 for Windows (GraphPad Software, La Jolla, USA).

## Results

### Bead morphology of the encapsulated bacteriophage preparations

**Fig. 2:**
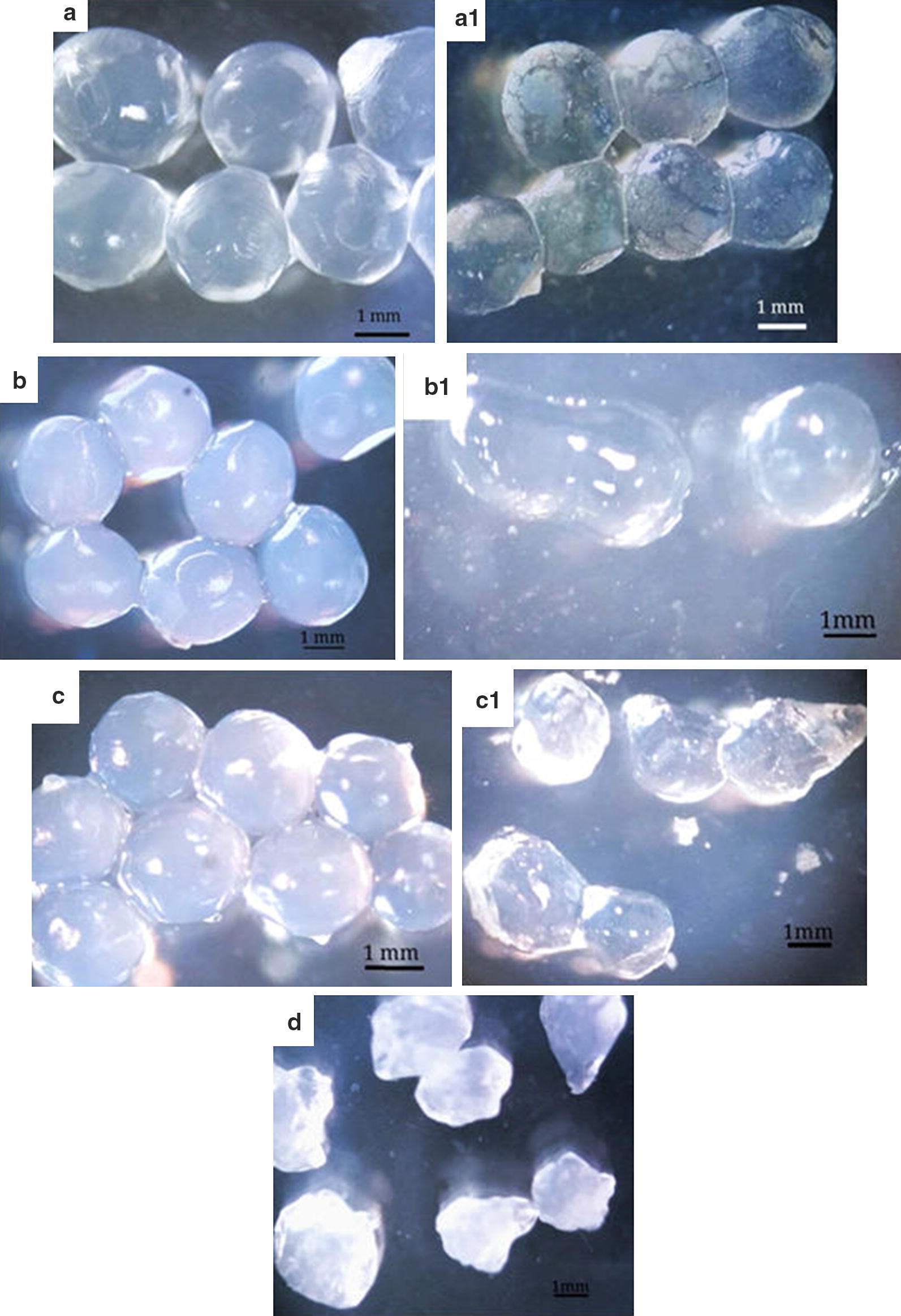
Optical micrographs of beads 1 (0.3% honey, 0.25% gelatin) in fresh form (**a**), beads 1 after 1-h incubation (**a1**), beads 2 (3% honey, 2.5% gelatin) in fresh form (**b**), beads 2 after 1-h incubation (**b1**), beads 3 (50 mM Tris-HCl pH 7.4) in fresh form (**c**), beads 3 after 1-h incubation (**c1**) and beads 4 (0.01% gelatin, 0.05% honey, 0.15 M NaCl and 10 mM MgSO_4_·7H_2_O) in fresh form (**d**), each bead was loaded with bacteriophage ZCEC5 in simulated intestinal juice

**Fig. 3:**
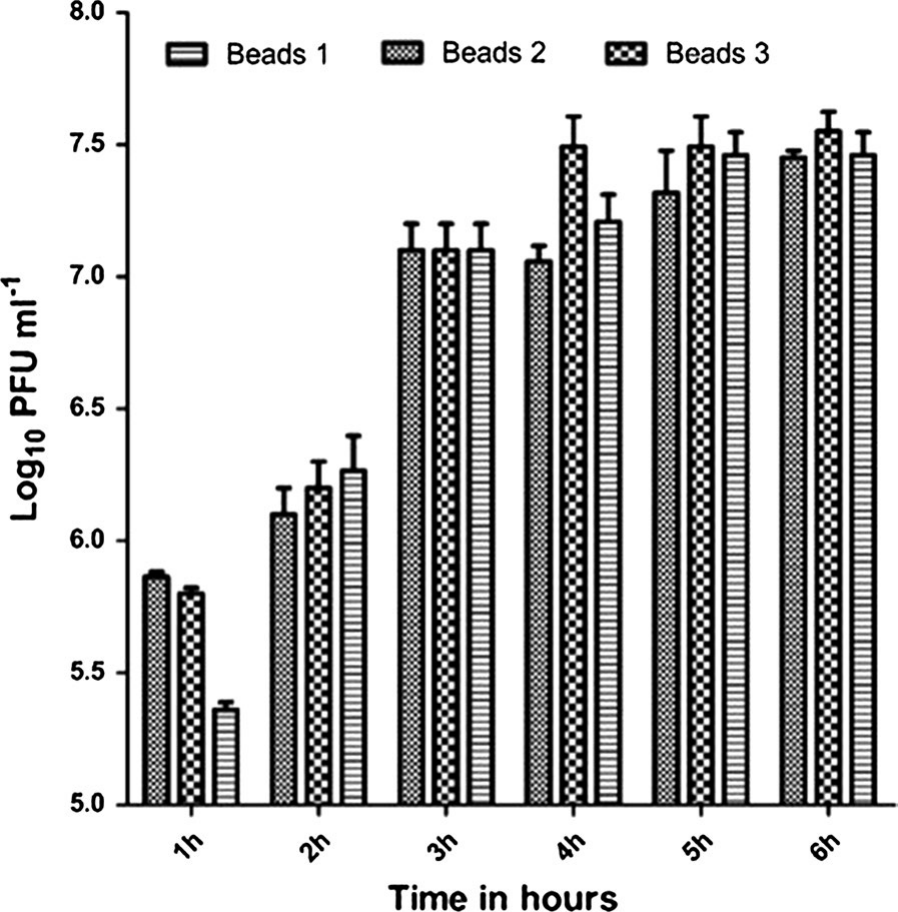
In vitro Log_10_ PFU mL^−1^ release of phages from chitosan–alginate capsules during incubation in gastrointestinal fluid for 6 h

The morphological characteristics of the ZCEC5 phage-encapsulated beads were determined by inverted microscopy. Beads 1, 2 and 3 appeared spherical shape with mean diameters of 2.38 ± 0.14, 2.8 ± 0.11 and 2.33 ± 0.12 mm, respectively (Fig. 2a–c). Bead preparation 4 (0.01% gelatin, 0.05% honey, 0.15 M NaCl and 10 mM MgSO_4_·7H_2_O) appeared non-uniform and irregular in shape (Fig. 3d), and was withdrawn from further experiments.

### Assessing leakage of encapsulated bacteriophages upon storage

To determine the retention and stability of the encapsulated bacteriophages, beads were stored in distilled water at 4 °C and samples collected every day for 8 days and after 8 weeks of storage. Over the course of the experiment, no phage release was observed under the storage conditions.

### Release rate of encapsulated bacteriophages under stimulated intestinal conditions

The bacteriophage release properties of the beads were measured after incubation in simulated gastrointestinal fluid (Fig. 3). The beads performed similarly producing titers in the range of 5.3 to 5.8 log_10_ PFU mL^−1^ after 1 h incubation and achieving 7.4 to 7.5 log_10_ PFU mL^−1^ after 5 h of incubation that approximates to full release of the matrix titer.

### Lytic activity of non-encapsulated and encapsulated bacteriophages

**Fig. 4:**
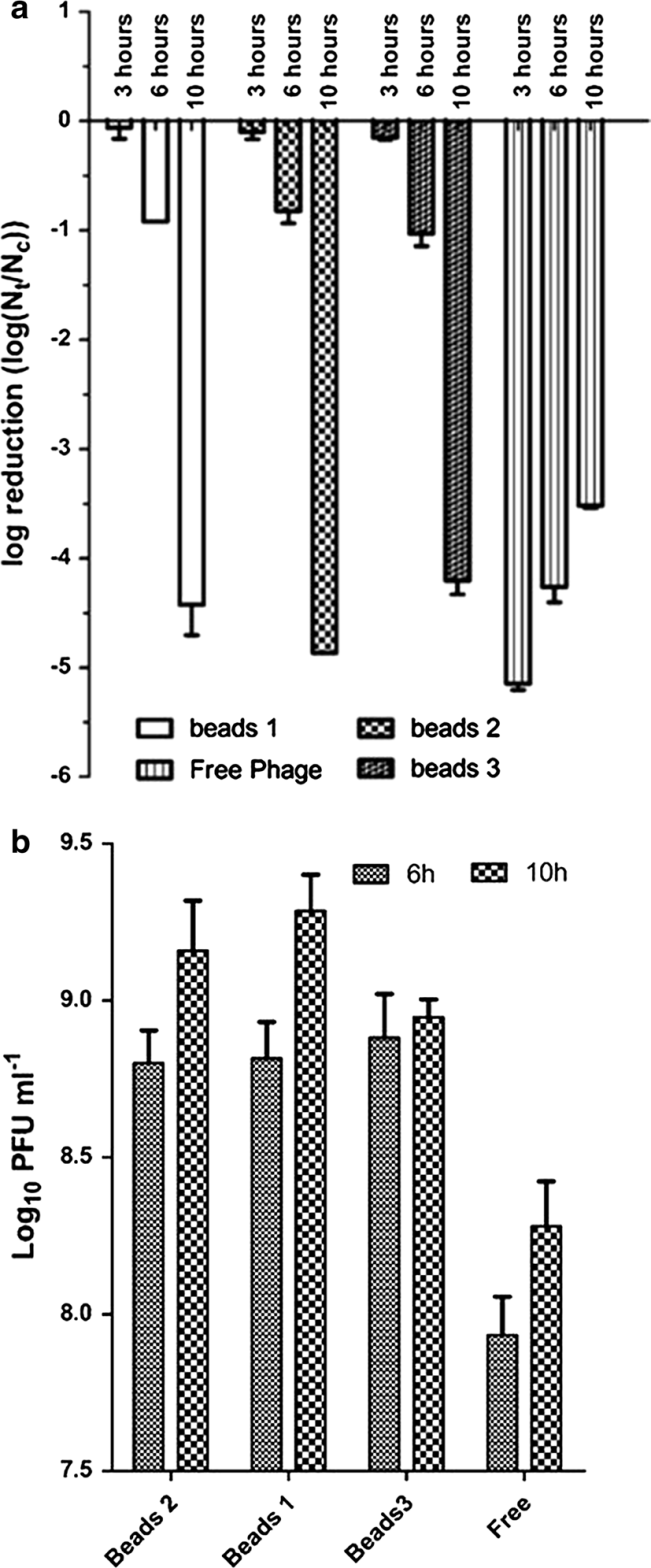
**a** Log_10_ reductions of *E. coli* O157:H7 incubated with encapsulated and non-encapsulated bacteriophages in gastrointestinal fluid at 37 °C for 6 h and 10 h. Nt stands for the number of *E. coli* O157:H7 after treatment with encapsulated and non-encapsulated bacteriophages at MOI = 1 and Nc represents the number of *E. coli* O157:H7 at the control state. All phage treatments produced significant falls in the viable count of *E. coli* O157:H7 (*p* value < 0.01). **b** Bacteriophage titers (Log_10_ PFU) of non-capsulated and encapsulated phages after infecting *E. coli* O157:H7 at MOI = 1 in gastrointestinal fluid for 6 h and 10 h

The lytic activities of non-capsulated and encapsulated bacteriophages were determined against *E. coli* O157:H7 NCTC 12900 over 3, 6 and 10 h in simulated intestinal conditions at MOI = 1 (Fig. 4a). Non-encapsulated ZCEC5 showed maximal reductions in the viable count of *E. coli* O157:H7 NCTC 12900 of 5.1 log_10_ CFU mL^−1^ after 3 h and declined upon increasing the incubation time under the simulated intestinal conditions. Conversely, bead-encapsulated bacteriophages exhibit a delay in the observed reduction of *E. coli* O157:H7 NCTC 12900 that is commensurate with the cumulative release of bacteriophage ZCEC5 over time. After 10 h maximal reductions for the bead-encapsulated treatments (4.2 to 4.8 log_10_ CFU mL^−1^) were comparable to that of free phage at 3 h (Fig. 4a). Host infection in gastrointestinal fluid lead to a 100-fold amplification of the bead-encapsulated phages over the initial titer of 7 log_10_ PFU mL^−1^ at 10 h, compared to tenfold recorded for free ZCEC5 phage infection (Fig. 4b).

### Acid and thermal acid stability of bead-encapsulated phage

**Fig. 5:**
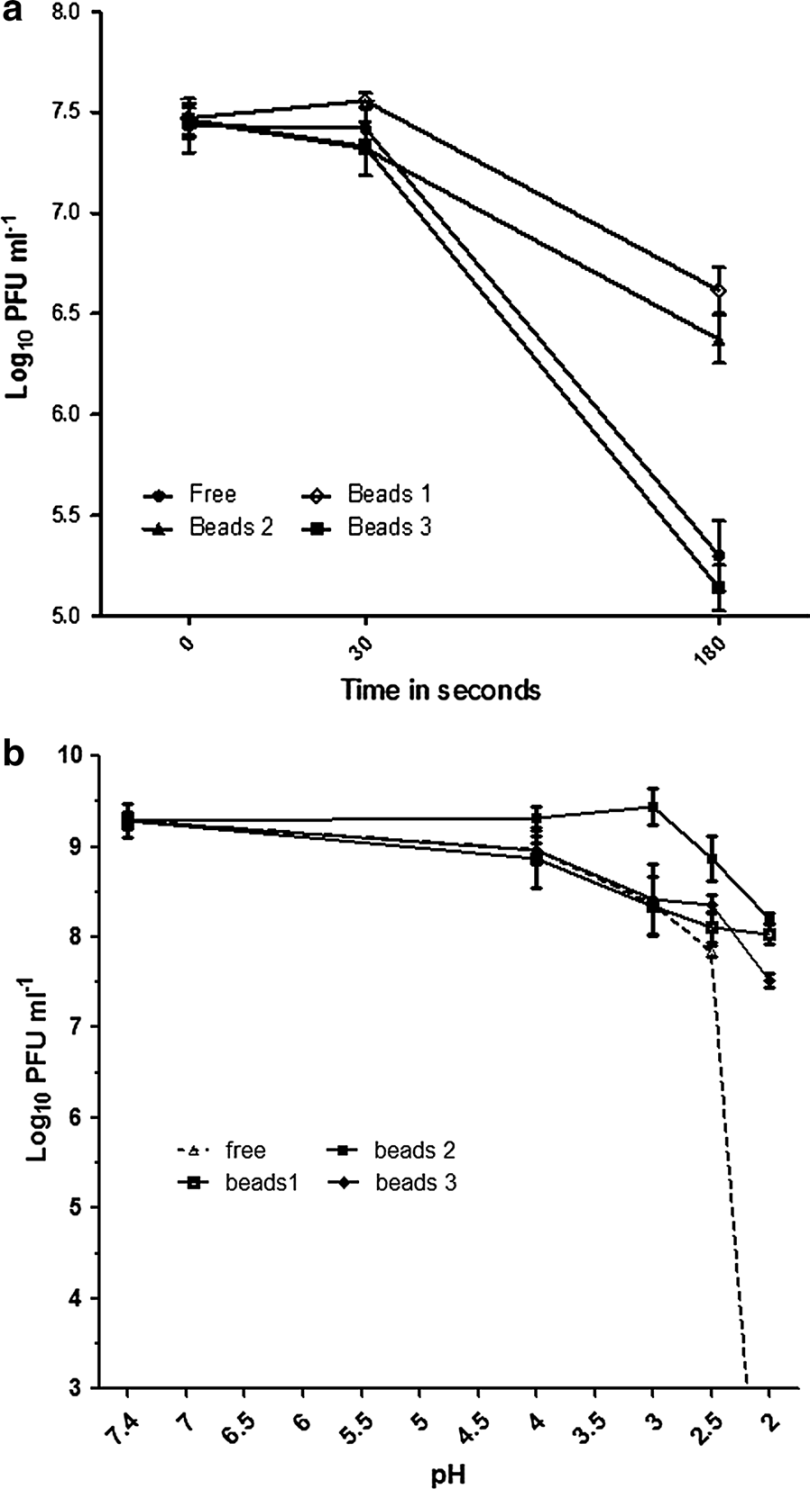
**a** The stability of non-encapsulated and encapsulated bacteriophages against high temperature (80 °C) for 3 min. **b** Low pH stability of the non-encapsulated and encapsulated bacteriophages 1 h at 37 °C. Limit of detection is < 10^3^ PFU mL^−1^

The stability of the bead-encapsulated bacteriophages in comparison to non-encapsulated bacteriophages were evaluated at acidic pH values, pH 2, 2.5, 3 and 4 over 1 h at 37 °C (Fig. 5b). The viability of non-encapsulated bacteriophages at pH 2 was measured after 30 s, 5 min and 10 min, where their titers were observed to decrease by 2 log_10_ PFU mL^−1^ after 30 s before falling below the detection limit (3 log_10_ PFU mL^−1^) after 10 min. The viability of the bead-encapsulated phages were tested after 1 h incubation at the pH indicated at 37 °C for 60 min in a dissolving buffer solution to release the encapsulated bacteriophages. Bead-encapsulation of bacteriophages has a protective effect against acid stress with approximately a 1 log_10_ PFU reduction observed at pH 2 compared to complete inactivation of the free phage. The matrix formulation of bead preparation 3 containing higher concentrations of glycerol and honey provided the greatest protection against low pH with no significant difference in the titer recovered post treatment at pH 3.

The role of each matrix component in conferring thermal protection was investigated by determining the phage titers released from the beads after 0.5, 1 and 3 min of heat treatment at 80 °C (Fig. 5a). Phage encapsulated in the matrix formulations of beads 1 and 2 containing honey and glycerol were more resistant to the heat treatment (titer reductions of 0.8 to 1 log_10_ PFU mL^−1^) than free phage (titer reduction 2.2 ± 0.22 log_10_ PFU) or the Tris-buffer based matrix of bead preparation 3 (titer reduction 2.3 ± 0.14 log_10_ PFU).

## Discussion

Ensuring the stability of bacteriophages is a key concern in the design of any phage therapy delivery method. Phage encapsulation is a promising technique that employs feed compatible materials that have no detrimental effect on phage activity. We demonstrate that bead-encapsulation can control the delivery of bacteriophage ZCEC5 in simulated gastrointestinal fluid and protect the phage from harsh conditions encountered in the stomach and intestinal tract to enable therapeutic delivery to farm animals. The simple protocol produced an efficiency of encapsulation that approached 100% and conferred increased acid and thermal stability comparable to previous reports of phage encapsulation (Ma et al. 2008; Dini et al. 2012; Tang et al. 2013; Colom et al. 2017). Bead-encapsulated bacteriophages showed excellent stability with no loss in phage titer when stored at 4 °C for 8 weeks. We have reduced the concentration of alginate to 1.5% compared to previous reports of 2–2.2% without leakage of phage from the matrix (Kim et al. 2015; Ma et al. 2008). Bacteriophage administered to farm animals must tolerate the acidic environment of the stomach. Under simulated intestinal conditions, chitosan–alginate encapsulated phages showed greater stability than the non-encapsulated phages (p < 0.01), with phage titer losses of 0.95–1.3 log_10_ PFU mL^−1^ after 1 h of incubation at 37 °C at pH 2 compared to non-encapsulated phage that were extremely sensitive to acidic conditions at pH 2. Although limited, the phage titer reductions observed suggest that the chitosan–alginate capsule does not prevent acid diffusion to the core of the capsule, a process that will contribute to exposure time dependent phage release in simulated intestinal solutions. These observations are consistent with those reported previously using phage preparations against *Vibrio vulnificus* (Koo et al. 2000). The incorporation of honey and gelatin in the matrices of bead preparations 1 and 2 increased their ability to protect the bacteriophage payload; a strategy based on reducing the rate of proton diffusion by increasing the viscosity of the bead matrix (Tyrrell 1981; Ma et al. 2012).

The controlled time-dependent release of bacteriophage ZCEC5 was achieved using the chitosan–alginate multilayer bead, which forms a cross-linked matrix that is preferable to fixing phages in gel networks (Anal and Stevens 2005; Colom et al. 2017). The pore size of the carbohydrate polymer shell is less than 200 nm (Andresen et al. 1977), which is smaller than the ZCEC5 phage size (223 nm) and ensures the encapsulated phages are retained. Controlled release alters the dynamics of phage infection to delay the delivery of the active phage and extend the period in which the host bacteria are lysed. The prolonged activity of the bead-encapsulated phage is in contrast to the action of free phage that exhibit a reduction in the ability to kill the host and increase phage titers with time. The extended time of delivery and lysis activity, have the potential to reduce the development of phage resistance.

In conclusion, this study demonstrates the efficient protective effect of core matrix materials in chitosan–alginate bead-encapsulated phage against inactivation by low pH, and to sustain bacteriophage release and lysis activity over time. Bead-encapsulation represents a simple inexpensive phage oral drug delivery system suitable for on farm applications directed to control the intestinal colonization of zoonotic and pathogenic bacteria. Further studies have the potential to combine nutritional and therapeutic components with phages to aid recovery.

## Data Availability

All data are available.
